# Aspects of Carbon Monoxide in Form of CO-Releasing Molecules Used in Cancer Treatment: More Light on the Way

**DOI:** 10.1155/2017/9326454

**Published:** 2017-02-13

**Authors:** Malamati Kourti, Wen G. Jiang, Jun Cai

**Affiliations:** Cardiff China Medical Research Collaborative (CCMRC), School of Medicine, Cardiff University, Heath Park, Cardiff CF14 4XN, UK

## Abstract

Carbon monoxide (CO) has always been recognised as a toxic gas, due to its higher affinity for haemoglobin than oxygen. However, biological studies have revealed an intriguing role for CO as an endogenous signalling molecule, a gasotransmitter. CO is demonstrated to exert many cellular activities including anti-inflammatory, antiapoptotic, and antiproliferative activities. In animal studies, CO gas administration can prevent tissues from hypoxia or ischemic-reperfusion injury. As a result, there are a plethora of reports dealing with the biological applications of CO and CO-releasing molecules (CORMs) in inflammatory and vascular diseases. CORMs have already been tested as a therapeutic agent in clinical trials. More recently, an increased interest has been drawn to CO's potential use as an anticancer agent. In this review, we will aim to give an overview of the research focused on the role of CO and CORMs in different types of cancer and expand to the recent development of the next generation CORMs for clinical application in cancer treatment.

## 1. Introduction

**Figure 1:**
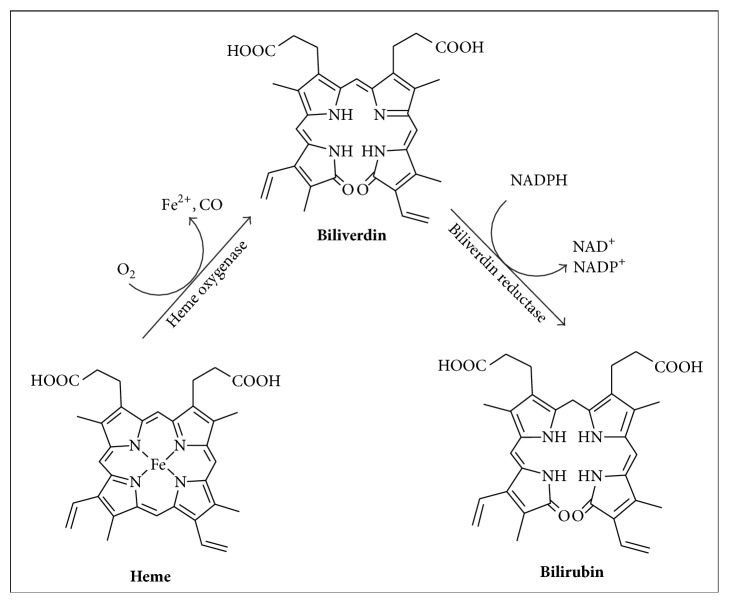
Oxidative conversion of heme. This figure depicts a summary of the formation of endogenous CO, carried out by a family of enzymes known as haem oxygenases (HOs), found as three isoforms: HO-1, HO-2, and HO-3.

Since 1949 when carbon monoxide (CO) was firstly identified as a natural metabolite in the exhaled air of healthy humans, it took a long time to be recognised as a molecular mediator. CO has been classified with other biologically active diatomic molecules, such as nitrogen oxide (NO) and hydrogen sulphide (H_2_S), as a gasotransmitter [1]. Naturally, CO is an inert gas, colourless, odourless, tasteless, and nonirritating. For decades, CO had the reputation of a “silent killer” owing to its more than 220 times stronger affinity for iron atoms in haemoglobin than oxygen, with the subsequent formation of carboxyhaemoglobin (HbCO). This binding significantly reduces the oxygen-carrying capacity of haemoglobin, consequently leading to tissue hypoxia and CO poisoning in humans [2]. Despite known as an environmental pollutant, CO is constantly produced in healthy humans endogenously [3]. The formation of endogenous CO is mainly carried out by a family of enzymes known as haem oxygenases (HOs), found as three isoforms: HO-1, HO-2, and HO-3 [4]. HO-1 and HO-2 catalyse the oxidative conversion of heme to ferrous iron, CO, and biliverdin, which is subsequently reduced to bilirubin by biliverdin reductase [5] (Figure 1). The function of HO-3 remains unclear, but it is viewed as a pseudogene derived from HO-2 transcripts [6]. HO-1, mainly found in spleen and liver, but also in vascular endothelial cells and smooth muscle tissues, is the only inducible isoform, increased as a result of several stimuli provoked by cellular stress. HO-2 and HO-3 are ubiquitously expressed in the brain, liver, and testes, with HO-2 being responsible for neurotransmission and regulation of vascular tone [2, 4, 5].

The endogenous actions of CO derive from its role as a physiological signalling molecule and affect many systems, such as the neuronal, cardiovascular, immune, respiratory, reproductive, and gastrointestinal systems [3]. CO exerts antiapoptotic, anti-inflammatory, and antioxidant effects, being able to maintain cell and tissue homoeostasis. It also acts as an antiproliferative and vasodilator agent and may be involved in tissue regeneration and strengthen the innate immune system [1, 3]. There is much evidence supporting the beneficial effects of CO in a vast array of diseases, such as cardiovascular disorders, sepsis and shock, cancer, acute and chronic rejection after organ transplantation, kidney and liver dysfunctions, haematological diseases, hypertension, inflammation, and neurodegeneration [2–4, 7]. All these conditions have been linked to abnormalities in the function of HOs and endogenous CO metabolism. However, the great number of diseases affected and potentially benefited by CO reflects the complexity of the CO-dependent signalling networks, which produce different responses according to the stimulus and type of tissue considered [2].

Although CO is suggested to be involved in many pathological conditions, the relevant research is only at an early stage, with many diseases yet to be explored towards their response to this interesting diatomic molecule. Since only few studies are focusing on the role of CO, along with biliverdin, bilirubin, iron, and ferritin, in carcinogenesis [8], this review aims to elucidate the role of this gasotransmitter in the form of CORMs, in tumorigenesis, along with angiogenesis and metastatic progression. We also want to highlight the development of CORMs, commonly used as alternatives to CO gas administration, as a type of “prodrug.”

## 2. Carbon Monoxide-Releasing Molecules (CORMs)

Although CO gas exerts a broad range of biological effects with many potential therapeutic applications, the lethality derived from CO effects on oxygen transport prevents the systemic CO application. Other practical issues include the global diffusion of CO, incapable of targeting specific cells or tissues, as well as the challenge of effective dose control. Therefore, a group of transition metal carbonyls or boranocarbonates, known as CO-releasing molecules (CORMs), which can release CO upon transformation becomes the best alternative to gas administration [2–5, 9, 10]. Since high concentrations of CO are proven to be cytotoxic via inhibition of the mitochondrial respiratory system [11, 12], CORMs offer a possible strategy for a local release of high amounts of CO for specific cytotoxicity only to the tumour region [1].

CORMs were first introduced as industrial catalysts and for purification purposes. They usually contain a transition metal core, such as manganese, ruthenium, or iron, surrounded by some carbonyl groups (CO) as coordinated ligands [5]. Both Schatzschneider and Zobi recently reviewed the potential trigger mechanisms for CO release from these compounds and categorized them into three groups [4, 13, 14]: (1) first group that releases CO due to ligand exchange reactions with the medium, (2) second group that needs a proper internal or external stimulus to induce the release, and (3) third group that explores differences in cellular microenvironments, such as enzyme expression or pH, to be used as alternative trigger mechanisms. These approaches could finally provide novel molecules with the ability of delivering tissue-targeted, internally triggered, and dose-controlled CO. Indeed, CORMs have shown a variety of activities, such as vasodilatory, anti-inflammatory, antiapoptotic, anti-ischemic, and cardioprotective activities, and have also been proven to regulate mitochondrial respiration [3].

Since 2008, a particular group of light-activated CORMs (also called photo-CORMs) has received most of the scientific interest. Under specific conditions, photoexcitation of the metal-carbonyl bond can lead to the dissociative loss of CO [5]. Photo-CORMs have been developed to meet certain criteria of all chemical compounds intended to be used as pharmaceutical drugs, which other CORMs failed to address [15]. Apart from a favourable pharmacokinetic behaviour concerning the properties of absorption, distribution, metabolism, and excretion (ADME), CORMs should also possess other characteristics, such as water solubility, biocompatibility, stability in aqueous aerobic media and air, low toxicity and quick detoxification of the M(CO)_*x*_ fragment, and traceable nontoxic metabolites [1, 4]. Once the bioavailability and optimal distribution of the photo-CORM are achieved, photochemical excitation of the metal-carbonyl bond could allow for controlling the timing, dosage, and location of the CO released. Until now, the primary challenge in the designing of new photo-CORMs is the selection of the ideal radiation wavelength at which they are activated. Currently, in vivo applications of the phototriggered CORMs may be limited to the sites of the body surface or the sites of the body closer to the surface [1, 4]. There are several reviews published on the matter of photo-CORMs, a quite comprehensive one being by Rimmer et al. [15].

**Figure 2:**
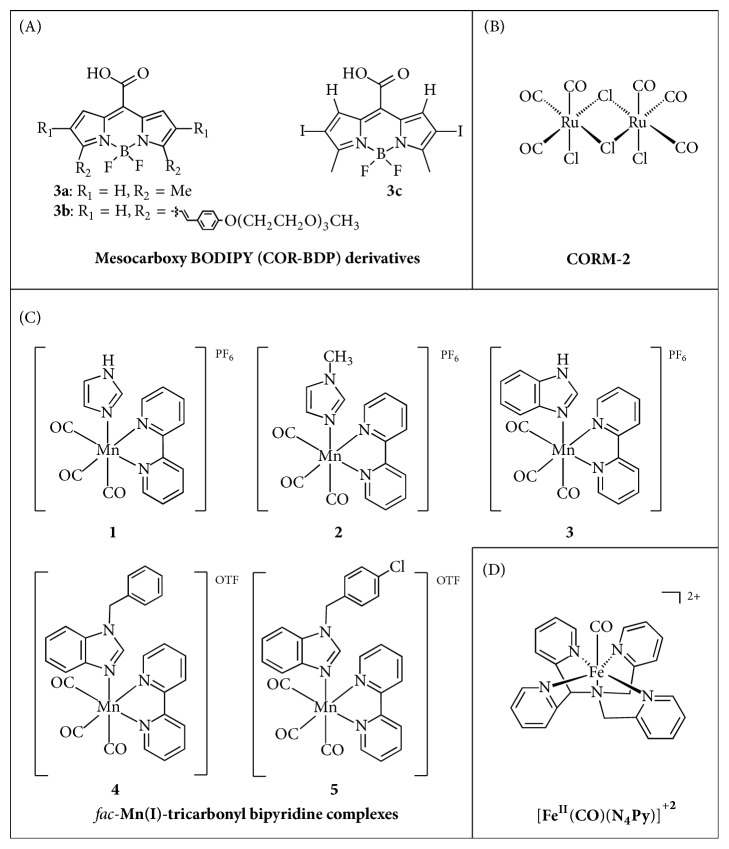
Structures of classic and novel CORMs.

A very interesting new generation of transition metal free CORMs based on boron-dipyrromethene (BODIPY) chromophores (named COR-BDPs) were synthesised by Palao et al. [16]. These molecules are activatable by visible-to-near-infrared (up to 730 nm) light. These wavelengths are highly desired, since they display a better penetration into biological tissues. Three structures of mesocarboxy BODIPY (COR-BDP) derivatives, 3a, b, and c, were reported (Figure 2(A)). Neither 3a and b nor their photoproducts displayed toxicity in in vitro experiments on hepatoblastoma HepG2 and neuroblastoma SH-SY5Y cell lines up to concentrations of 100 *μ*M. However, after CO release studies, the researchers found that 3c, unlike 3a and b, undergoes a photoreaction that does not lead to CO production. The main advantage of these COR-BDPs is that the BODIPY scaffold allows for fine-tuning of the physicochemical properties of the final molecules, such as optical properties or aqueous solubility. Moreover, simple structural modifications are feasible; therefore a rapid development of improved CORM molecules could be achieved. The therapeutic potential of COR-BDPs remains to be investigated in further studies [16].

As previously mentioned, CORMs regulate intracellular pathways, which play significant roles in inflammation, apoptosis, and cellular proliferation [7]. The preclinical efficacy and the structures of a broad range of early and newly synthesised CORMs have been recently reviewed [1, 3, 4, 7, 17]. It is pretty evident that numerous diseases can be affected by these compounds, indicating the complexity of CO-signalling networks. Compared to many reports with the biological applications of CORMs in inflammatory and vascular diseases, microbial infections, and organ transplantations [2, 3, 5, 7, 10], little interest has been drawn to the potential use of CO and CORMs as anticancer agents. In this review, we aim to summarise the research that has been done so far to elucidate the effects of CO and CORMs on different subtypes of cancer.

## 3. CORMs and Cancer Therapy

The potential application of CO and CORMs in cancer therapy is still controversial [1, 18]. The molecular targets and specific signalling pathways affected by CO remain unclear, which often leads to contradicting reports [19–21]. The most possible scenario is that CO can stimulate opposite effects in various biological systems. CORMs have been studied for their influence on cell proliferation, apoptosis, and angiogenesis, primary processes involved in cancer initiation and progression, and the conclusion until now is that this effect is variable and seems to be cell-type specific [18, 22]. So, the main purpose of this review is to gather the experimental data available to date on the potential effects of CO and CORMs on various cancer subtypes. In this review, organometallic carbonyl complexes which are incapable of releasing CO are excluded, since the point of interest is the effect of CO gas or CORMs on cancer.

### 3.1. Breast Cancer

Being one of the most common subtypes of cancer, breast cancer was immediately considered a potential target disease for CO and CORMs. In that scope, Lee et al. [23] found that CO, as a byproduct of HO-1, could attenuate heat shock protein 90 (HSP90) activity and reduce the expression of its client proteins, which participate in the six hallmarks of cancer. The novelty of this study lied upon the inhibitory mechanism elucidation, since they demonstrated that treatment with CORM-2 up to 100 *μ*M and for 24 h (Figure 2(B)), as a source of CO, increased wild type (WT) p53 expression in MCF-7 breast cancer cells, while it reduced mutant p53 levels in MDA-MB-231 breast cancer cells. Mutant p53 is an oncoprotein addicted to HSP90 and, together with other HSP90 client proteins, including cyclinD1 and Akt, that were also shown to be reduced by CORM-2 indicated a CO-induced downregulation of HSP90 activity. This result revealed a potential therapeutic advantage of CORM-2, since targeting HSP90 may deal with multiple molecules at the same time and it seems that CO plays an important role in the modulation of this chaperone [23]. Carrington et al. [24] synthesised two manganese carbonyl complexes, namely,* fac-*[MnBr(azpy)(CO)_3_] (1) and* fac-*[Mn(azpy)(CO)_3_(PPh_3_)](ClO_4_) (2), which were characterized as photo-CORMs. The two CORMs released CO upon irradiation with low power visible light. The anticancer potential of the former was evaluated via MTT assay on MDA-MB-231 breast cancer cells, under the control of visible light. Upon irradiation, a dose-dependent eradication of the cancer cells was observed, with a concentration of 75 *μ*M killing nearly 40% of the colony. Therefore, this complex offered anti-breast cancer activity through unique light-controlled CO delivery [24]. The same group [25] also applied a novel technique for drug delivery, utilizing Al-MCM-41 mesoporous silica nanoparticles (MSNs) loaded with a newly synthesised photo-CORM, namely,* fac*-[Re(CO)_3_(pbt)(PPh_3_)](CF_3_SO_3_), (ReCO), for the selective delivery of the photo-CORM to breast cancer cells. The endocytosis of the [Re-CO]@Al-MCM-41 MSNs within MDA-MB-231 breast cancer cells provides the opportunity for trackable CO delivery, due to a “turn-off” effect of a systematic decrease in luminescence intensity upon UV irradiation. Moreover, they tested the anticancer activity of the endocytosed particles after light-induced CO release on MDA-MB-231 cells via MTT assay. The results indicated an 80% eradication of the cancer cells at a concentration of 0.25 mg/mL of the nanoparticles, a much stronger effect than that after the application of the photo-CORM alone, due to the extravasation of nanosized particles occurring in a tumour-selective manner [25]. In a later study, Carrington et al. [26] designed a fluorescent manganese carbonyl complex, namely,* fac*-[MnBr(CO)_3_(pbt)], which was sensitive to visible light and displayed an increase in fluorescence upon CO release as a “turn-on” photo-CORM. This complex showed a dose-dependent inhibition of MDA-MB-231 breast cancer cells, by reducing their viability approximately 50% at the highest concentration of 100 *μ*M, under visible light illumination. The results suggested that this manganese photo-CORM was able to deliver CO to biological targets upon irradiation with visible light, provoke CO-induced apoptosis in human breast cancer cells, and offer the ability to track CO delivery within the cells, because of the highly fluorescent pbt included in the complex [26]. Finally, Üstün et al. [27] synthesised* fac*–manganese (I) tricarbonyl bipyridine complexes with imidazole and benzimidazole substituents (Figure 2(C)), which were shown to release CO upon UV light irradiation. The anticancer potential of these complexes was investigated against a human invasive ductal breast carcinoma (MCF-7) cell line and all of them displayed promising IC_50_ values of low micromolecular range. However, since the aim of the study was to inhibit breast cancer cell proliferation upon UV irradiation due to the release of CO from the designed compounds, the same in vitro experiment was repeated with a 10-minute irradiation at 365 nm and the different IC_50_s were reported. It seems that compounds 1, 3, and 5 exert cytotoxic effect on MCF-7 cells, and UV irradiation does not enhance cytotoxicity of these compounds. On the contrary, compounds 2 and 4 express a UV-dependent cytotoxicity, following the principles of photo-CORMs design. Therefore, compounds 2 and 4 may provide photodynamic agents for anticancer therapy with controlled CO release profiles [27].

### 3.2. Prostate Cancer

Prostate cancer is the most common type of cancer in males. Jackson et al. [28] described the synthesis of a new photo-CORM [Fe^II^(CO)(N_4_Py)](ClO_4_)_2_ (Figure 2(D)) that released CO after UV light irradiation, which was tested for its anticancer effect on PC-3 prostate cancer cells. Their data clearly demonstrated a concentration-dependent eradication of the cancer cells, with the greatest effect at 10 *μ*M. Also, the authors found that this complex possesses a suitable ligand structure for peptide attachment [28]. In addition, Pierri et al. [29] generated a novel photo-CORM, a water-soluble rhenium complex, that is,* fac*-[Re^I^(bpy)(CO)_3_(thp)]^+^. This complex, as well as its photoproduct, did not show significant toxicity against human prostatic carcinoma cells PPC-1 in the dark up to 100 *μ*M. However, the experiments demonstrated a significant aggregation in the cytoplasm, but not within the nucleus [29]. Further experiments are needed though to evaluate the possible anticancer activities of* fac*-[Re^I^(bpy)(CO)_3_(thp)]^+^.

### 3.3. Colon Cancer

**Figure 3:**
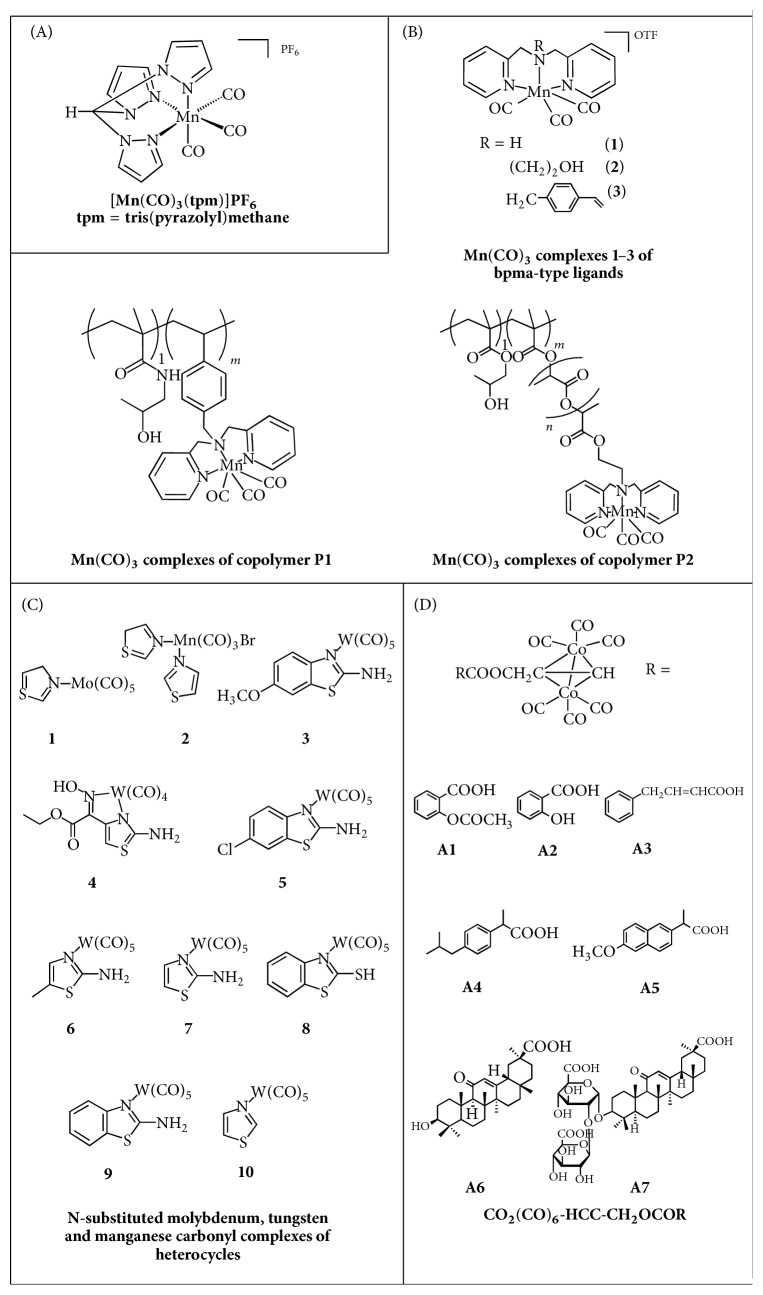
Structures of novel CORMs.

Niesel et al. [30] reported a novel manganese complex, namely, [Mn(CO)_3_(tpm)]PF_6_ (Figure 3(A)), that could be activated by UV light and used as a potential photo-dynamic-therapy (PDT) agent. According to cellular uptake studies, this complex was suggested to enter the cells by passive diffusion rather than active transport and thus may be useful for cellular delivery of CO. The differential cytotoxicity of the complex with or without irradiation was tested on HT29 colon cancer cells via the crystal violet assay. Upon irradiation, the complex inhibited cell growth comparable to the well-known anticancer agent 5-fluorouracil, reducing cell biomass nearly 30% compared to the control at a concentration of 100 *μ*M. Therefore, this complex may serve as a PDT agent against colon cancer [30]. Brückmann et al. [31] synthesised a series of complexes exploring the idea of linking photo-CORMs to HPMA-based copolymers, generating functionalized copolymers (Figure 3(B)). The cytotoxicity of these complexes was tested on Hct116 human colon carcinoma cells and compared to that of CORM-2. Only two of these compounds showed toxicity, with IC_50_ values close to 50 *μ*g/mL for both. However the researchers concluded that it was not a direct result of CO release, because the toxicities of the two complexes remained the same with or without irradiation. This study demonstrated the feasibility of developing ligand functionalized polymers as carrier systems for Mn(CO)_3_-based CORMs for the passive delivery of these CORMs to tumour tissues or other sites [31].

### 3.4. Cervical Cancer

The manganese complex* fac-*[MnBr(azpy)(CO)_3_], previously mentioned in the breast cancer section, was also tested for inhibitory effects on a cervical cancer cell line, that is, HeLa cells [24]. This complex exhibited similar anticancer effects on the cervical cells as those against the breast cancer cell line upon irradiation with visible light. The complex caused certain morphological changes to the cervical cancer cells, indicating an apoptotic cell state, suggesting a potential use as an anticancer agent for breast or cervical cancers, with the advantage of a favourable light-controlled CO release [24]. Hu and colleagues [32] designed a series of N-substituted molybdenum, tungsten, and manganese carbonyl complexes containing various heterocycles (Figure 3(C)) which were tested for cytotoxicity on HeLa cells. Apart from complex 4, the rest of series complexes exert modest inhibitory effects on HeLa cells, without significant cytotoxicity. Among them, complex 3 was the most potent, with an IC_50_ of 37.22 *μ*M [32]. Interesting are also the relatively slow CO-releasing cobalt-containing CORMs (Figure 3(D)) designed by Gong et al. [33]. Appropriate MTT assays showed that the slow CO-releasing CORMs could inhibit the proliferation of HeLa cells with IC_50_s less than 120 *μ*M, reflecting their potential anticancer activity. The data of animal experiments, further, showed low LD_50_ values via the oral acute toxic class method (ATC), in the range of 5000 mg/kg. The mechanism of action was further studied, revealing a concentration-dependent apoptotic effect of complexes 1 and 6, mainly in the later stage. Finally, the authors proved that these complexes could block cell cycle in the G2/M phase, inhibiting division and proliferation, and increasing intracellular reactive oxygen species (ROS) concentration, possibly through CO and cobalt ions released after CO loss. Hence, they hypothesised that the antiproliferative activity of the synthesised compounds resulted from the binding of CO to proteins of the mitochondrial electron transfer chain leading to an intracellular ROS level increase, as well as the release of cobalt species that can bind to endogenous substrates interrupting cell cycle and provoking apoptosis [33].

### 3.5. Pancreatic Cancer

Vítek et al. [34] first indicated that CO, in the form of CORM-2 or CO gas, exerted a potential antiproliferative effect on a panel of human pancreatic cancer cell lines, in a dose-dependent manner. CO inhibited the proliferation of the PaTu-8902 cells and induced apoptosis in the CAPAN-2 pancreatic cancer cells at a concentration of 50 *μ*M. CORM-2 treatment led to an increase in the survival rate of in vivo pancreatic cancer models with a significant decrease in the tumour volumes at 35 mg/kg/day. CORM-2 was also able to substantially affect de novo angiogenesis via suppressing Akt phosphorylation, a significant contributor to cancer neovascularisation. The authors concluded that exogenously applied CO, either in the form of CORM-2 or as direct CO gas exposure at 500 ppm, could inhibit pancreatic tumour growth and tumour neovascularisation and prolong survival and thus could act as an inhibitor of pancreatic carcinogenesis [34]. Schwer et al. [35] on the other hand identified the inhibitory effects of CORM-2 on components of the translational machinery and global protein synthesis of pancreatic stellate cells (PSCs). Being a common histopathological feature in pancreatic cancer, fibrosis has been shown to be significantly affected by both processes of translation and protein synthesis. CORM-2 prevented serum-induced eEF2 dephosphorylation, in a time-dependent manner and inhibited the PI3K-Akt-mTOR signalling pathway in PSCs, independently of the ERK1/2 signalling pathway, at concentrations of up to 100 *μ*M. Moreover, CORM-2 treatment at the highest concentration used resulted in a 51% reduction in the protein synthesis of PSCs, which combined with elevated intracellular calcium and cAMP levels indicated that CORM-2 could inhibit the translational machinery, regardless of HO-1 induction. The anti-inflammatory and antiproliferative effects of CORM-2 could hence be via repressing global protein synthesis [35].

### 3.6. Skin Cancer

The antiphotocarcinogenic properties of CO were established in another study by Allanson and Reeve [36]. They explored whether CO signalling has an anti-skin cancer function. Their results suggested that topically applied CORM-2, in the form of lotions of 250 and 500 *μ*M and as a source of CO, produced a moderate inhibition of early tumour appearance, increased the elimination of established tumours, and inhibited the development of malignant and locally invasive large tumours. All the effects of CORMs were dose-dependent, even though not always following the expected pattern. CORM-2 also appeared to significantly reduce average tumour multiplicity [36].

### 3.7. Lung Adenocarcinoma

HO-1 was shown to inhibit lung adenocarcinoma cell growth via generation of CO [8]. CO gas treatment, instead of CORMs, could cause a time-dependent inhibition in the frequency, proliferation, and size of the tumour nodules in a lung tumour Kras mouse model, if administered at 250 ppm for 1 h/day. Also, CO gas increased the levels of mtTFA indicating mitochondrial stress. CO gas could not enhance the sensitivity of A549Rho^o^, the mitochondria-depleted A549 cells, to doxorubicin, suggesting that CO gas may rapidly enhance mitochondrial activity of cancer cells, resulting in metabolic exhaustion and cellular collapse under intense oxidative stress and thus tumour regression [8]. A549 cells were derived from the lung tumour Kras mouse model.

### 3.8. Lymphoma and Acute Myeloid Leukaemia

**Figure 4:**
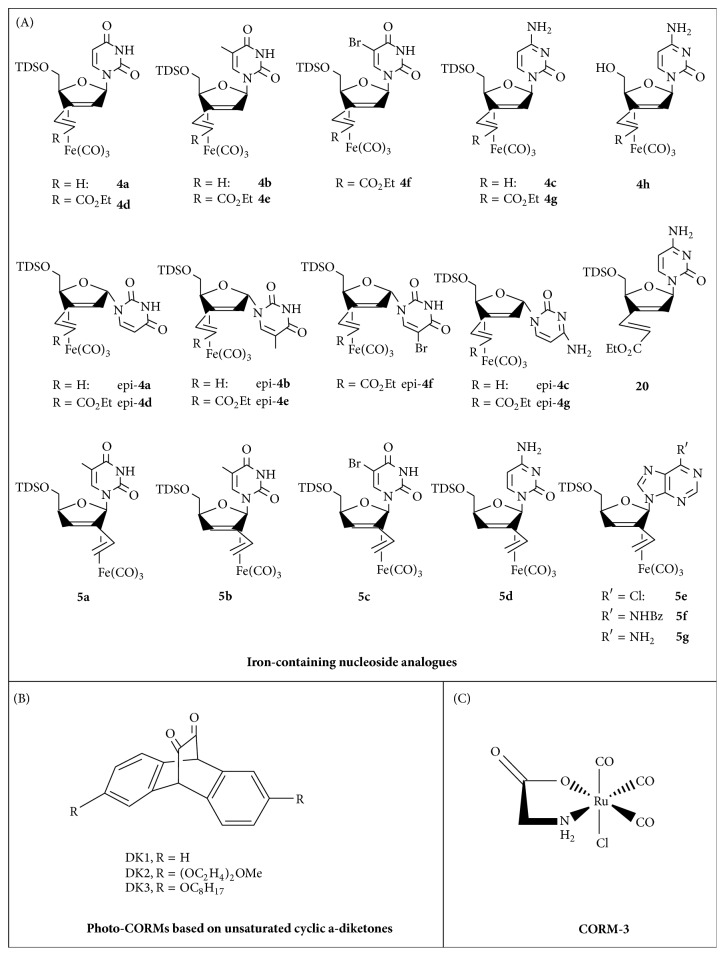
Structures of first-generation and novel CORMs.

Loureiro et al. [37] developed a novel nanocarrier system loaded with 6 *μ*M CORM-2. The system was tested on a lymphoma cell line (A20 cell line), as well as in vivo in BALB/c mice bearing subcutaneous A20 lymphoma tumours. To take advantage of the ability of the nanocarriers to affect the pharmacokinetics and drug distribution to different tissues, PEGylated and folic acid- (FA-) functionalized BSA nanoemulsions were generated, which could specifically deliver CORM-2 into FA receptor-positive cancer cells. The authors found that CORM-2 could halt A20 cell proliferation, possibly via HO-1 signalling, without affecting endogenous reactive oxygen species (ROS) levels. These nanoemulsions loaded with CORM-2 also led to a decrease in cell viability in vitro, measured via MTT assay. In the in vivo experiments, treatment with these nanoemulsions containing CORM-2 resulted in a 46% reduction in the tumour size, as well as enhanced survival rates [37]. The study by Schlawe et al. [38] demonstrated a new class of cytostatic, apoptosis-inducing agents. The authors generated some iron-containing nucleoside analogues (Figure 4(A)) and tested them for their cytotoxic activity against a lymphoma B cell line, namely, BJAB cells. The cytosine derivatives were the most potent complexes with IC_50_s of 20–30 *μ*M. Iron-containing nucleosides appeared to be able to induce concentration-dependent apoptosis, based on the observation of blebbing, as well as DNA fragmentation after treatment with 20 *μ*M. The antileukemic potency of the new nucleosides was also evaluated, with one particular complex efficiently inducing apoptosis in an ex vivo DNA-fragmentation assay involving primary leukemic lymphoblasts. However, the mechanism of action needs to be further elucidated but may involve CO release as well [38]. Finally, a new series of organic photo-CORMs based on unsaturated cyclic *α*-diketones (Figure 4(B)), encapsulated in Pluronic F127 micelles at 407 *μ*M, were also reported by Peng et al. [39]. The novel organic CORMs could initiate a photoreaction triggered by visible light instead of UV light, required by most organometallic photo-CORMs for CO release. The authors tested these micelle-encapsulated organic photo-CORMs for effects on leukemic (KG1) cell proliferation and viability by flow cytometry. Their results showed that the viability of the cells was not altered by the photo-CORMs or the anthracene based fluorophore. Also, no photodamage was observed. Therefore, this study revealed a new approach in the designing and synthesis of new photo-CORMs, beyond their potential use as anticancer agents, which can prove well suited for possible targeted delivery of CO to biological systems [39].

## 4. Mitochondrial Respiration and Glucose Biotransformation

Toxic effects of CO are due to its high affinity for the reduced iron-heme in haemoglobin, crucial for the delivery of oxygen (O_2_) to tissues. Thus, CO can compete with O_2_ for the binding site of heme on the mitochondrial cytochrome c oxidase, one of its central targets, and become harmful for cellular respiration [40]. CO has also been shown to increase mitochondrial biogenesis and ATP and ROS generation, which also affects cellular behaviour [4, 7, 8]. For instance, increased ROS production can lead to the upregulation of peroxisome proliferator-activated receptor *γ* (PPAR*γ*) and hypoxia-inducible factor 1*α* (HIF-1*α*) [7].

A proof-of-concept study by Lo Iacono et al. [11] suggested that CORM-3 (Figure 4(C)) could prove a novel regulator of mitochondrial respiration, via the liberated CO, that could be useful for diseases where mitochondrial uncoupling and metabolism are targeted as therapeutic options. Interestingly, low concentrations of CORM-3, 2–50 *μ*M, stimulated a concentration-dependent increase in O_2_ consumption during state 2 respiration, which could be blocked by a CO scavenger, whereas a decrease was observed for state 3 respiration rate. However, higher concentrations of CORM-3, 50–100 *μ*M, were able to reduce state 2 respiration. The iCORM-3 (unable to release CO) could not exert the same effects, suggesting that the uncoupling effect and inhibition of mitochondrial respiration caused by CORM-3 are most likely due to CO rather than the rest of the molecule. Furthermore, CORM-3 was also shown to accelerate O_2_ consumption and inhibit complex IV individually, at the highest concentration tested (100 *μ*M) [11]. Takano et al. [41] found that there should be an alternative mechanism for cellular energetics to maintain ATP in the CO-exposed cells. The authors examined the impact of CO on cell bioenergetics and glucose utilization through the fluxome analysis with liquid chromatography-mass spectrometry (LC-MS/MS), on U937 cells. Their results showed that CORM-2 at 100 *μ*M, as a source of CO, regulated glucose biotransformation and suppressed glycolysis, results that need further examination though [41].

## 5. Effects of CORMs on Cancer-Related Conditions

### 5.1. Cardiotoxicity of Doxorubicin

CORMs have also been considered against other cancer-related pathological conditions, most commonly side effects caused by other anticancer drugs. In that scope, Soni et al. [42] demonstrated the potential protective properties of CO, in the form of CORM-2, against the doxorubicin- (DXR-) induced acute cardiac failure-cardiotoxic effects. The mouse model treated with CORM-2 (30 mg/kg) for a long period before DXR administration manifested the less impaired heart function compared to that treated with DXR alone at a dose-dependent manner (3–30 mg/kg, i.p.). These cardioprotective effects were probably derived from the CORM-2-induced reduction in the serum levels of creatine kinase (CK) and lactate dehydrogenase (LDH), leading to further attenuation of oxidative stress and cardiomyocyte apoptosis. In addition, the study suggested that CORM-2 protection against cardiotoxicity may involve the activation of the HO-1 signalling pathway, by increasing HO-1 mRNA expression in DXR-treated mouse hearts. Thus CORM-2 should be considered a therapeutic prevention for DXR-induced cardiotoxicity [42].

### 5.2. Nephrotoxicity of Cisplatin

A notorious side effect of cisplatin (CP-a widely used anticancer drug) is nephrotoxicity. Tayem et al. [43] examined CORM-3 as a potential protective agent against nephrotoxicity using both in vitro and in vivo assays. CORM-3 (1–50 *μ*M) appeared to ameliorate the CP-induced nephrotoxicity and renal dysfunction through protecting both renal tubule epithelial cells (LLC-PK1) and kidneys against the structural and functional impairment induced by CP. In vivo administration of CORM-3 (10 mg/kg, i.p.) prevented CP-induced acute renal failure and helped in preserving kidney morphology and preventing weight loss. Their study indicated a novel use of CORMs, as sources of endogenous CO, as alternative agents to prevent CP-induced nephrotoxicity [43].

### 5.3. Radiotherapy-Induced Secondary Cancer

From another point of view, Tong et al. [44] studied the potential employment of low-concentration CO combined with radiotherapy, which may lead to protecting normal tissues beyond the irradiated area against the radiation-induced bystander effects (RIBE), preventing secondary cancer. By using the RIBE system, an increase in chromosome aberration in proliferating bystander Chinese hamster ovary (CHO) cells, the authors successfully showed that low concentrations of exogenous CO, delivered by CORM-2 at 14 *μ*M, could effectively inhibit the RIBE-induced cell proliferation and chromosomal abnormality (MN and NPB) [44].

## 6. CORMs and Angiogenesis

The effects of CO gas and CORMs on tumour angiogenesis are again highly controversial. Different studies report contradicting results based on the types of tumours studied. They conclude that these agents have either a pro- or antiangiogenic effect based on the type of cells involved.

Choi et al. [45] suggested a stimulatory effect of CO on angiogenesis, mainly mediated by elevated intracellular ROS levels in endothelial cells. This effect is directly linked to the inhibitory activity of CO on mitochondrial complex IV, involved in the respiratory system of these organelles [1, 45]. The authors also reported that CO could induce proangiogenic factors, such as VEGF and IL-8, and suppress antiangiogenic mediators. The same group [46] in another study reported that CO could induce VEGF expression through hypoxia-induced factor *α* (HIF-1*α*) activation and stabilisation. Similarly, CORM-2 could provoke a paracrine-style of proangiogenic activity. The media derived from astrocytes treated with CORM-2 could stimulate human umbilical vein (HUVEC) cells to proliferate, migrate, and form tubes, an action that could be inhibited by a VEGF-neutralizing antibody. CORM-410, a novel oxidant-sensitive CORM, was reported to promote angiogenesis via CO-mediated induction of HO-1 and phosphorylation of p38 MAP kinase. In addition, CORM-401 increased VEGF and IL-8 levels, both highly involved in angiogenesis [47]. Fang et al. [48] found that CORM-2 could increase tumour blood flow, thus having a proangiogenic effect on solid tumours. Other papers reported similar results that CO could induce VEGF expression or enhance its angiogenic activity [49–51].

In contrast, there are reports suggesting that both HO-1 and CO could have variable functions, based on the different cellular microenvironments [48, 49, 51, 52]. Some of these reports described an antiangiogenic effect of HO-1 and CO on cancer cells [19, 34, 52]. Ahmad et al. [19] showed that CORM-2 (50 *μ*M) could inhibit VEGF-related cell proliferation and migration via inhibition of VEGF-R2 and protein kinase B (Akt) phosphorylation in HUVEC cells. In pancreatic cancer, CO inhibited endothelial proliferation and reduced microvascular density of xenon cancer models [34]. The study by Skrzypek et al. [53] indicated that CORM-2 treatment also led to the inhibition of angiogenic mediators in non-small-cell lung cancer. Moreover, as previously mentioned for breast cancer, CORM-2 was proved to downregulate the HSP90 client protein activity [23] and inhibit the synthesis of MMP-9 [54].

Loboda et al. [18] have comprehensively discussed the controversial effects of CO gas or CORMs on angiogenesis. The authors thoroughly examined both potential types of effects that may be modulated by heme oxygenase-1 (HO-1) and CO regarding angiogenesis. First they make the point that (1) induction of angiogenesis is mainly supported by an increase in growth factors synthesis and activation, an inhibition of the differentiation of cancer stem cells, and a boost in VEGF expression and release and (2) angiogenesis may be attenuated via a decreased synthesis of angiogenic mediators, an inhibition on the phosphorylation of key kinases, and a metabolic switch from glycolysis towards the pentose phosphate pathway. In this review as well as all others, CO is suggested to influence tumour angiogenesis in opposite ways. It has been reported to increase the synthesis of growth factors and improve the viability of endothelial cells and may be linked to cancer initiating cells. CO could also increase VEGF synthesis and other proangiogenic factors. However, in other cancers, high HO-1 expression and CO, often in the form of CORMs, were correlated with impaired tumour growth. Indeed, as mentioned above antiangiogenic activities of CO were reported in various studies, as well as antiproliferative effects and inhibition of xenotransplanted tumours. CO was also correlated with downregulation of HSP90 client protein activity and inhibition of the synthesis of MMP-9. Therefore, it is obvious that the perplexity of the potential role of HO-1/CO still remains to be elucidated, and it is urgently needed to further examine how this role is implicated in tumour development and angiogenesis. Only in this way could CORMs be considered for more personalised therapies, potentially as additional therapeutic agents for cancer patients [18].

## 7. Designing of New CORMs

So, the question now is, what is next? There is a growing interest in developing novel CO-based therapeutics, and as the understanding of CO biology increases every day we expect to see CORMs becoming a new class of therapeutics against various diseases soon [55]. There have been many obstacles for CORMs to develop as clinically useful agents, though. The main problem is obviously solubility, but researchers have been able to address this problem successfully. Nevertheless, there are many other issues to be dealt with, such as stability in air and water, biocompatibility, how to ensure low or no toxicity of the metal-fragment left after the release of CO, and favourable pharmacokinetics [1, 2, 4, 7]. Apart from the properties of absorption, distribution, metabolism, and excretion (ADME) required for all drug-like substances, the most important feature of effective CORMs is to be able to reach the appropriate disease sites where they can release CO via a certain trigger or stimulus in the proper amount and time [1, 2, 10]. Moreover, the half-life of the CORM in the circulation and the release of CO are crucial for the desired therapeutic effect. The design of new CORMs should be based on finding suitable ligands capable of tuning the half-life of the CO release [2, 4, 13].

The structure of a CORM contains three parts: the metal core, the ligand (inner) coordination sphere or CORM sphere, and the drug (outer) sphere. The ligand sphere determines the kinetics and stoichiometry of the CO release, given a proper metal core, whereas the drug sphere should provide the CORM with desired characteristics, such as favourable ADME and tissue-specific targeting elements [1, 13]. Comprehensive reviews of conceptualising novel CORM structures are introduced by many researchers [4, 10, 13]. The most important aspects of designing new CORMs include the number of releasable CO groups, the kinetics of the CO release, and the trigger mechanisms required for initiation. The chemical versatility of these compounds is their most significant advantage over CO gas. The appropriate targeting ligands conjugated to the metal-carbonyl scaffold allow for their spatial distribution in the various body fluids and tissues, even in different cell subpopulations [4, 13].

As for the special class of photo-CORMs, additional properties are required, such as photoreactivity at specific wavelengths where the penetration depth is optimal, that may allow for total control of the delivery and activation of these compounds. This could only be achieved by suitable metal and coligand combinations [15, 55, 56].

## 8. Conclusion

Further development of metal-based CORMs is on its way and we expect to see more advanced and well-targeted molecules arising in the near future. In this perspective, it would be even possible to select the proper CORM for the therapeutic action needed, according to its CO-releasing profile and pharmacokinetic behaviour. Overall, appropriate designing and synthesis of novel molecules could prove invaluable towards a more personalised cancer treatment and a reduced drug load for cancer patients [4].
